# Detecting trabecules of the systemic right ventricle during quantification yields better correlation with flow measurement derived data

**DOI:** 10.1186/1532-429X-17-S1-Q95

**Published:** 2015-02-03

**Authors:** Attila Tóth, Hajnalka Vágó, András Temesvári, Hajnalka O Bálint, Csilla Juhász, Barbara C Jánosa, Ferenc I Suhai, György Balázs, Kálmán Hüttl, András Szatmári, Béla Merkely

**Affiliations:** 1 Heart and Vascular Center, Semmelweis University, Budapest, Hungary; 2 Gottsegen György Institute of Cardiology, Budapest, Hungary

## Background

During the past few years commercial solutions became available to deal with ventricular trabecules. It has been already shown, that the new algorithms have a significant impact on all parameters measured during routine evaluation (EF: ejection fraction, ESVi: end systolic volume index, EDVi: end diastolic volume index, SVi: stroke volume index, Mi: mass index). The effect of trabecules become especially important in case of pressure overloaded right ventricle. It has been also published, that cardiac ultrasound (ECHO) tend to underestimate volumes and overestimate ejection fraction when compared to cardiac magnetic resonance (CMR) as a gold standard.

## Methods

43 male (21.26 ± 5.28 years of age) and 10 female (19.60 ± 4.06 years of age) patients after Senning procedure were included in the current analysis. MR instrument: Philips Achieva 1.5T. Evaluation software: Medis QMass MR 7.6. Systemic ventricular parameters were assessed on short axis stack of slices (thickness: 8mm, spacing: 0mm) both using regular 3D and the new MassK algorithms (technical details published in the literature). The two methods were compared in a pair-wise manner. Moreover, breath-hold, 2D, through-plane flow measurements were also performed on the aortic valve. Flow analysis was carried out using: Medis QFlow 5.6. Ventricular SV and cardiac output (CO) measured by each quantification methods were correlated to the flow derived CO and SV values as a reference. Only 39 patient were included in these calculations due to exclusion of 14 patients because of various reasons relevant to the analysis.

## Results

Comparison of the two ventricular evaluation methods yielded similar results to other groups engaged in this topic. Namely: ESVi, EDVi and SVi turned out to be significantly lower, while EF tend to become significantly higher when using the new trabecular quantification. Not surprisingly, the ventricular mass increased significantly as well in this patient group. SV and CO measured by both ventricular evaluation methods were in close correlation with the flow derived SV and CO values (regular vs trabecular: 0.8301 vs 0.8298 for CO, and 0.7945 vs 0.7978 for SV). Bland-Altman analysis demonstrated, that the regular algorithm have a remarkable bias - overestimating the flow data (regular vs trabecular bias [CI lower - upper]: 1.58 l/min [-0.15 - 3.30] vs -0.01 l/min [-1.49 - 1.47] for CO, 23.03 ml [-3.54 - 49.60] vs -1.61 ml [-25.04 - 21.83] for SV). Trabecular thresholding method have a much lower bias, giving results more equivalent to the reference flow data.

## Conclusions

Using the new trabecular quantification method introduce significant differences in all measured parameters for the systemic right ventricle patient group - in accordance with the current literature, while also bringing the results closer to the flow determined values, suggesting that it's superior. Therefore it probably means another step in making CMR as an established gold standard to become more perfect.

## Funding

None.

**Figure 1:**
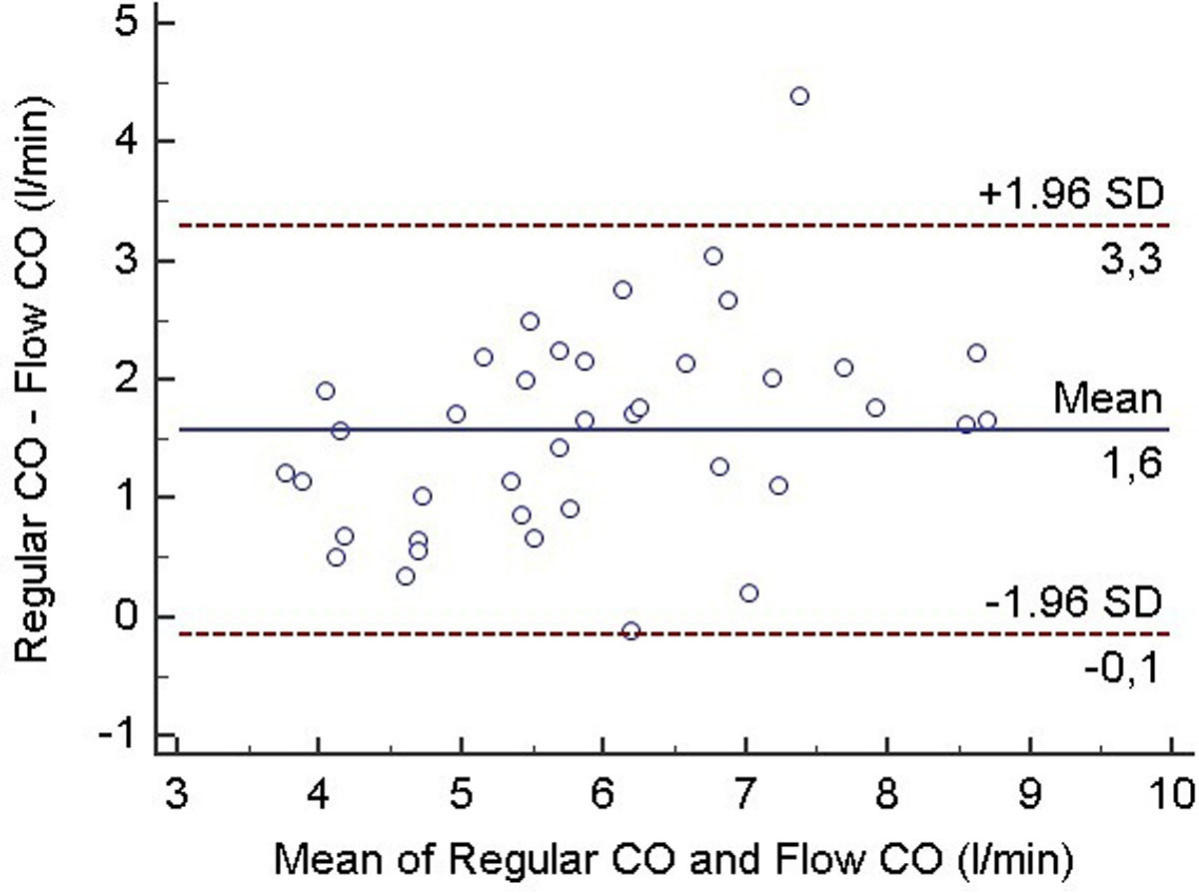
Large bias for regular evaluation against flow data

**Figure 2:**
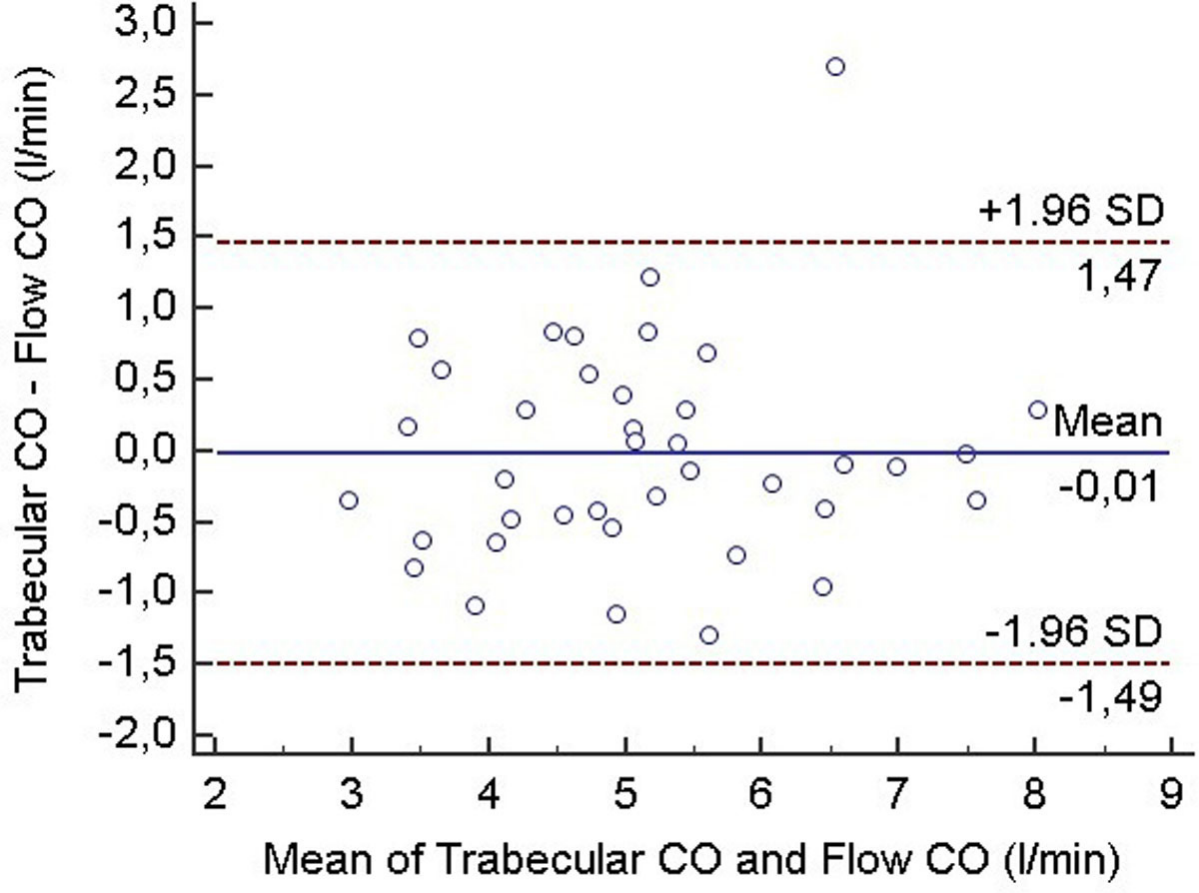
Just a small bias when comparing trabecular algorithm derived values vs flow results

